# Effects of a School-Based Nutrition, Gardening, and Cooking Intervention on Metabolic Parameters in High-risk Youth: A Secondary Analysis of a Cluster Randomized Clinical Trial

**DOI:** 10.1001/jamanetworkopen.2022.50375

**Published:** 2023-01-10

**Authors:** Jaimie N. Davis, Matthew J. Landry, Sarvenaz Vandyousefi, Matthew R. Jeans, Erin A. Hudson, Deanna M. Hoelscher, Alexandra E. van den Berg, Adriana Pérez

**Affiliations:** 1 Department of Nutritional Sciences, College of Natural Sciences, University of Texas at Austin, Austin; 2 Stanford Prevention Research Center, School of Medicine, Stanford University, Palo Alto, California; 3 Bellevue Hospital Center, Division of General Pediatrics, Department of Pediatrics, New York University School of Medicine, New York; 4 Health Equity Alliance, The Health Management Academy, Arlington, Virginia; 5 Michael & Susan Dell Center for Healthy Living, Department of Health Promotion and Behavioral Sciences, The University of Texas Health Science Center at Houston, School of Public Health, Austin Campus, Austin; 6 Michael & Susan Dell Center for Healthy Living, Department of Biostatistics and Data Science, The University of Texas Health Science Center at Houston, School of Public Health, Austin Campus, Austin

## Abstract

**Importance:**

Although school-based gardening programs for children have consistently been shown to improve dietary behaviors, no cluster randomized clinical trial (RCT) has evaluated the effects of a school-based gardening intervention on metabolic outcomes.

**Objective:**

To evaluate the effects of a school-based gardening, nutrition, and cooking intervention (Texas Sprouts) on changes in metabolic outcomes in elementary schoolchildren.

**Design, Setting, and Participants:**

This study was a secondary analysis of a cluster RCT, conducted over 3 years from 2016 to 2019, at low-income elementary schools with majority Hispanic students in the greater Austin, Texas, area. Data were analyzed from January to August 2022.

**Interventions:**

Texas Sprouts was 1 school year long (9 months) and consisted of (1) Garden Leadership Committee formation; (2) a 0.25-acre outdoor teaching garden; (3) 18 student gardening, nutrition, and cooking lessons taught by trained educators throughout the school year; and (4) 9 monthly parent lessons. The delayed intervention was implemented the following academic year and received an identical intervention.

**Main Outcomes and Measures:**

The following measures were obtained at baseline and postintervention (9 months): demographics via survey; measured height, weight, and body mass index parameters; and glucose, insulin, homeostatic model assessment of insulin resistance, and a lipid panel via an optional fasting blood draw.

**Results:**

Sixteen elementary schools were randomly assigned to either Texas Sprouts intervention (8 schools) or to delayed intervention (control, 8 schools). A total of 3302 children (aged 7-12 years) were enrolled in Texas Sprouts, and fasting blood samples were obtained from 1104 children (or 33% of those enrolled) at baseline. The final analytic sample included 695 children (307 boys [44.17%]; mean [SE] age, 9.28 [0.04] years; 480 Hispanic children [69.02%]; 452 [65.03%] eligible for free or reduced lunch) with complete demographic data and baseline and postintervention (9-month) fasting blood draws. Compared with control schools, children from Texas Sprouts schools had a 0.02% reduction in mean hemoglobin A_1c_ (95% CI, 0.03%-0.14%; *P* = .005) and a 6.40 mg/dL reduction in mean low-density lipoprotein cholesterol (95% CI, 3.82-8.97 mg/dL; *P* = .048). There were no intervention effects on glucose, insulin, homeostatic model assessment of insulin resistance, or other lipid parameters.

**Conclusions and Relevance:**

In this cluster RCT, Texas Sprouts improved glucose control and reduced low-density lipoprotein cholesterol in high-risk youth. These findings suggest that elementary schools should incorporate garden-based interventions as a way to improve metabolic parameters in children.

**Trial Registration:**

ClinicalTrials.gov Identifier: NCT02668744

## Key Points

**Question:**

Does a school-based gardening, nutrition, and cooking intervention affect changes in metabolic outcomes in elementary schoolchildren?

**Findings:**

In this secondary analysis of a cluster randomized clinical trial of 695 children, a gardening, nutrition, and cooking intervention resulted in reductions of 0.02% for hemoglobin A_1c_ and 6.40 mg/dL for low-density lipoprotein cholesterol levels.

**Meaning:**

These findings suggest that school-based gardening interventions may improve metabolic parameters in children.

## Teaser

This secondary analysis of a cluster randomized clinical trial evaluates the effects of a school-based gardening, nutrition, and cooking intervention on changes in metabolic outcomes in elementary schoolchildren.

## Introduction

The prevalence of childhood obesity in the US has increased over the last 4 decades, from 5% in 1978 to 19.3% in 2018.^1^ The Hispanic ethnic group represents the second fastest-growing minoritized population in the US, comprising 19% of the US and almost 40% of the Texas population.^2^ In Texas, 66% of adults and 45% of children (aged 7-9 years) have overweight or obesity,^3,4^ with the highest proportions among Hispanic individuals. Low socioeconomic status has been associated with an increase in overweight or obesity.^4^ Hispanic children are also more likely than non-Hispanic White children to develop obesity-related metabolic diseases, such as metabolic syndrome and type 2 diabetes.^5^ The prevalence of these illnesses could be reduced by increasing fruit and vegetable (FV) intake.^6^ Research^7,8^ has linked FV intake to reductions in metabolic syndrome, visceral fat, and type 2 diabetes risk in children from minoritized racial and ethnic groups. Most US children do not meet the recommended daily FV intake, and intake is lowest among low-income children and those with obesity.^9^ Therefore, evidence-based interventions are needed to improve FV intake and reduce obesity-related cardiometabolic diseases in low-income children from minoritized racial and ethnic groups.

In the past decade, many studies^10,11,12^ have consistently shown that school garden–based interventions can improve FV intake and dietary-related psychosocial variables in children. Wang and colleagues^10^ found that fourth and fifth grade students with the most exposure to a school-based gardening intervention increased their mean preference and intake of FV by one-half cup a day. A randomized clinical trial (RCT)^11^ showed that fourth grade students who received 16 weeks of nutrition education alone or nutrition education plus gardening significantly improved FV intake, compared with controls, but only the group exposed to gardening retained gains 6 months later. However, most of the aforementioned studies were conducted with non-Hispanic White children from middle income schools. In a cluster RCT, the Texas!Grow!Eat!Go! study^12^ showed that children receiving the school-based gardening intervention showed increases in nutrition knowledge, vegetable preference, and vegetables tasted compared with children at control schools. In a pilot RCT^13^ with 4 elementary schools, a 12-week after-school gardening intervention, called LA Sprouts, led to increased vegetable and dietary fiber intake and reductions in body mass index (BMI; calculated as weight in kilograms divided by height in meters squared) *z *scores and waist circumference. We recently conducted a cluster RCT^14,15^ and showed that a gardening intervention, called Texas Sprouts, which was taught during school hours over the course of 1 school year, resulted in increased vegetable intake and improved academic performance in intervention vs control schools; however, no change in adiposity or obesity measures was seen.

Most of the school-based interventions that have examined the effects of RCTs on metabolic outcomes have used a multicomponent approach where nutrition, physical activity, and behavior modification have been taught.^16,17,18^ To date and to our knowledge, no cluster RCT has been conducted to assess the effects of an in-school garden-based intervention on metabolic health outcomes. Therefore, the goal of this study is to evaluate the effects of the Texas Sprouts compared with control on changes in metabolic outcomes in elementary schoolchildren. The hypothesis was that children in the Texas Sprouts intervention compared with control will have improvements in glucose control and lipids and reductions in insulin and insulin resistance.

## Methods

### Study Design and Participants

The study protocol is shown in Supplement 1, and a detailed description of Texas Sprouts study design and main outcomes is provided elsewhere.^14,15^ This study was conducted over 3 years from 2016 to 2019. Texas Sprouts was a school-based cluster RCT with 16 elementary schools that were randomized to either intervention group: Texas Sprouts intervention (8 schools) or control (delayed intervention; 8 schools). The intervention was implemented in 3 waves over 3 years (2016-2019). The study statistician (A.P.), who was blinded to the identity of the schools, implemented the randomization and allocation of the schools. All schools met the following inclusion criteria: (1) majority Hispanic children (>50%); (2) majority of children (>50%) participating in the free and reduced-price lunch (FRL) program, which represents a low-income population; (3) location within 60 miles of central Austin, Texas; and (4) no existing garden or gardening program. All third to fifth grade students and parents at the recruited schools were contacted to participate via information tables at back-to-school events, flyers sent home with children, and teachers making class announcements in the fall after the garden had been built at the school. All recruitment materials were available in both English and Spanish. Although all children in third to fifth grade from participating schools received the lessons as part of their in-school curriculum, children and parents had to provide written informed consent to participate in the evaluation measurements. The study was approved by The University of Texas at Austin’s internal review board. This secondary analysis follows the Consolidated Standards of Reporting Trials (CONSORT) reporting guideline for RCTs.^19^

### Intervention

At each intervention school, garden leadership committees were formed composed of interested stakeholders, such as teachers, parents, community members, school staff, and children. With the help of the committee members, gardens were built in every intervention school in the spring before the academic year, approximately 4 months before baseline measurements.

Full-time experienced and trained nutrition and garden educators taught 18 one-hour Texas Sprouts lessons separately to each third to fifth grade class throughout the school year as part of their normal school day. The following are some of the broad nutrition concepts that were included in the curriculum: (1) healthy cooking and preparation of FV (ie, low in sugar and fat); (2) making nutritious food choices in different environments; (3) eating locally produced food; (4) low-sugar beverages made with fresh FV; (5) health benefits of FV; (6) how to eat healthfully in food desert neighborhoods (ie, neighborhoods lacking easy access to shops selling FV); and (7) food equity and community service. The curriculum also covered a broad range of horticultural and environmental education topics, including science process skills, observation, taking measurements, and problem-solving through both group and individual learning experiences. Every lesson included either a garden taste test (7 lessons) or a cooking activity (11 lessons). Every lesson also included sampling of different *aguas frescas*, which are flavored or infused water with no added sugar. Curriculum content and recipes were culturally tailored to Hispanic individuals and included recipes like vegetable quesadillas, corn and black bean salad, and juicy jicama salad. The garden and nutrition educators also taught monthly 60-minute Texas Sprouts lessons to the parents, for a total of 9 lessons, throughout the school year. Every lesson was also mapped on Texas Essential Knowledge Standards for science, math, language arts, health, and social studies.

The control schools received a delayed intervention (identical intervention as described above) in the year after completion of the posttesting for that wave. Baseline and postintervention measurements occurred for the control parents and children within the same period as the intervention schools.

### Outcome Measurements

#### Anthropometry and Demographics

Data were collected on children and parents at baseline (within the first month of the beginning of the academic school year) and postintervention (within the last month of the academic school year) at the school sites. Height was measured using a free-standing stadiometer (Seca) mounted against the wall, to the nearest 0.1 cm. Weight was assessed with the Tanita Body Fat Analyzer (model TBF 300). BMI and BMI percentiles were determined using Centers for Disease Control and Prevention age-specific and sex-specific values.^20^ Children were asked questions about their age, grade, and sex on a survey. Parents were asked to complete a questionnaire packet, which included information regarding their child’s race and ethnicity and participation in the FRL program. Race and ethnicity were assessed in this study because these variables have been linked to metabolic outcomes in youth in the literature.

#### Blood Collection

Blood draws were optional, and children who opted not to participate in the blood draw could still participate in all other Texas Sprouts evaluations and programming. Eligible children and their families received flyers and text message reminders about the optional blood draw and were instructed to come fasting, having nothing to eat or drink other than water after midnight. Blood draws were conducted over a 1-week period at each school and took place before the start of the school day or on Saturday mornings. Blood samples were collected by certified phlebotomists in a private room at the schools. Children were asked 3 times if they were fasting before the blood draw, twice during the check-in process and once by the phlebotomist conducting the draw. Blood samples were placed on ice immediately after being drawn. Children received a $20 incentive for participation in the blood draw, and parents were incentivized to have their children participate in the blood collection by receiving a free diabetes screening.

Directly following collection, whole blood was placed on ice and transferred to the laboratory on the University of Texas at Austin campus, where glucose was measured using a Glucose 201 analyzer (HemoCue America). Glycated hemoglobin A_1c_ (HbA_1c_) assays using DCA Vantage Analyzer (Siemens Medical Solutions) were performed on whole blood. The remaining blood was centrifuged, aliquoted, and stored at –80 °C. Samples were transported on dry ice to Baylor College of Medicine to assess insulin, cholesterol, and triglycerides. Insulin was evaluated using an automated enzyme immunoassay system analyzer (Tosoh Bioscience, Inc). Homeostatic model assessment of insulin resistance (HOMA-IR) was calculated according to the following formula: HOMA-IR = fasting glucose in millimoles per liter × fasting insulin in microunits per milliliter / 22.5. Total cholesterol, high-density lipoprotein (HDL) cholesterol, and triglyceride levels were measured using Vitros chemistry DT slides (Ortho Clinical Diagnostics Inc); low-density lipoprotein (LDL) cholesterol was calculated using the Friedwald equation.^21^ Of note, HbA_1c_ assays were initially not included in the protocol and were added after the first wave because of the higher than expected prediabetes rates from the fasting plasma glucose (FPG) values. The suggestion to add HbA_1c_ came from the physicians and scientists who served on the data safety monitoring board. Therefore, HbA_1c_ values are available for waves 2 and 3 only, representing 10 schools (5 intervention schools and 5 control schools).

Parents were contacted immediately by our study physician if their child’s FPG and HbA_1c_ values were in the diabetic range (FPG value of ≥126 mg/dL [to convert to millimoles per liter, multiply by 0.555] and/or HbA_1c _value ≥6.5% [to convert to proportion of total hemoglobin, multiply by 0.01]).^22^ For those children in the prediabetic range (FPG value of 100-125 mg/dL and/or HbA_1c _value of 5.7%-6.4%),^22^ results were sent home with the children in a sealed envelope and the families were recommended to follow up with their physician or a free health clinic.

### Sample Size

The sample size was estimated to test the effects of the intervention on blood glucose, with a power of 80% using a type I error of α = .05, a 2-sided test, and assuming equal allocation between the 2 groups.^23,24^ The variance (σ2) within schools, and the intracluster correlation coefficient used change data from children who completed the pilot LA Sprouts study.^25^ It was estimated that 6 schools each with 60 children per school who participated in blood draws were needed to detect the effect size of a decrease in FPG of 2.13 mg/dL. Two additional schools per group were included in case a school decided to withdraw participation. For these reasons, a total sample size of 16 schools was used for this study.

### Statistical Analysis

 Data were analyzed from January to August 2022. Study data were collected and managed using REDCap software version 12.2.11 (Research Electronic Data Capture). Children who completed the baseline and postintervention blood draw (ie, subsamples A and B) were used for the complete case analyses. Of note, multiple imputations were run in the main outcomes study for clinical outcomes (vegetable intake, body composition, blood pressure, and BMI parameters),^15^ but could not be run for blood samples as models would not converge; therefore, the analyses were run using complete case data. In addition, HbA_1c_ data were missing for wave 1 because that assay was not added until wave 2. Differences in demographic and adiposity characteristics were compared between the sample without baseline and postintervention blood draws and subsamples A and B using χ^2^ (for categorical variables) and independent tests (for continuous variables). Generalized weighted linear mixed models with the identity link for continuous variables were used to test differences in metabolic parameters between the intervention and the control estimates, with schools as clusters fixed for their intervention effect and children nested within schools. Data were analyzed using SPSS statistical software version 28 (IBM) and a 2-sided type I error level of α = .05 was used as the threshold for statistical significance.

## Results

The Figure shows the flow of participants through the Texas Sprouts study and which children completed the optional blood samples at preintervention and postintervention. Of the 4239 eligible children at the 16 schools, 3302 (78%) consented to be in the study and 3137 (74%) completed baseline clinical measures (height, weight, and BMI parameters) and child surveys. Approximately 2876 parents (92%) completed baseline surveys. A total of 1104 consented children (33%) completed the optional baseline blood draw. A total of 695 children who participated in the baseline blood (63%) draw also gave blood at the postintervention follow-up. Therefore, there were 2442 children who completed baseline clinical and survey measures who did not complete preintervention and postintervention blood draws. Blood glucose, insulin, HOMA-IR, and lipid tests were run on all available preintervention and postintervention blood samples (subsample A, 695 children), but there was not enough blood drawn or the blood hemolyzed in several samples; thus, insulin and HOMA-IR data are missing for 12 children, and lipid data are missing for 14 children in subsample A. Subsample B included all 457 children with complete HbA_1c_ at preintervention and postintervention from 10 schools.

**Figure.**
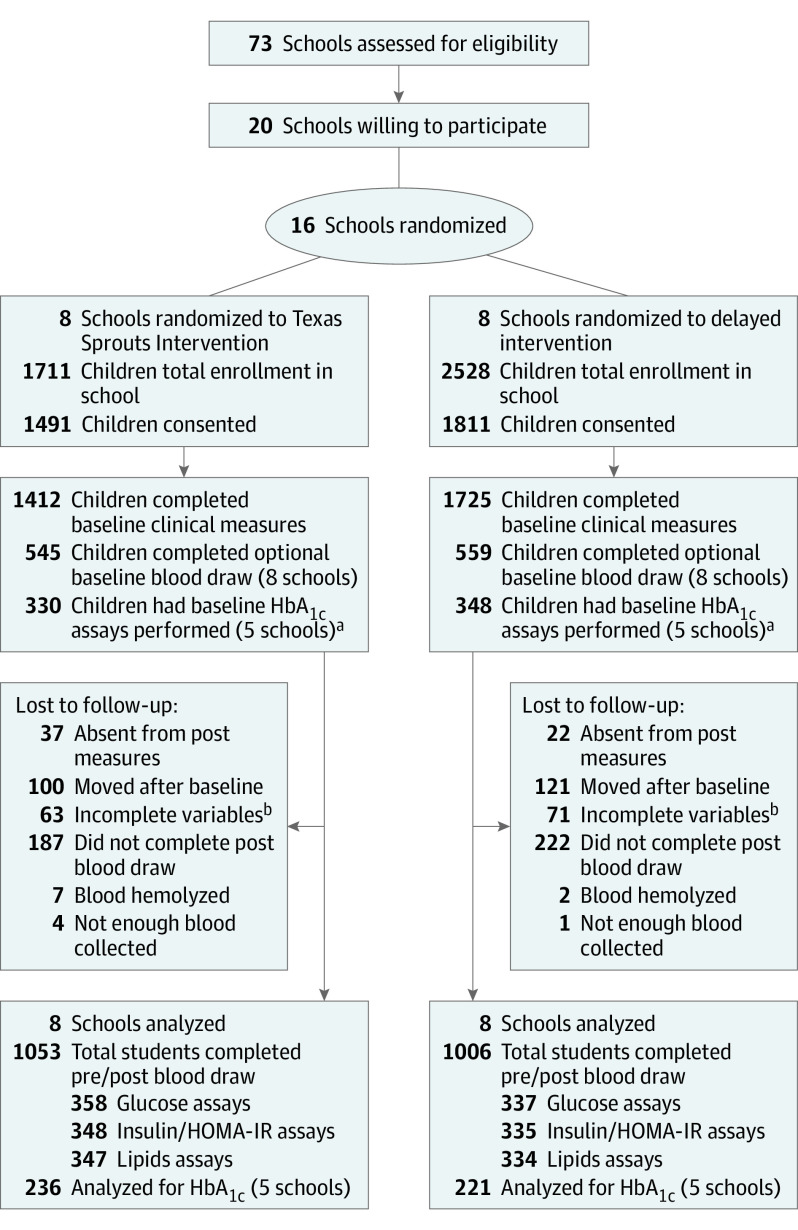
Study Enrollment Flowchart HbA_1c_ indicates hemoglobin A_1c_; HOMA-IR, homeostatic model assessment of insulin resistance. ^a^Prediabetes rates were higher than expected for fasting plasma glucose at wave 1; therefore, our data safety monitoring board recommended we add an HbA_1c_ test on whole blood at time of collection, which is why HbA_1c_ is available for only 10 schools and 2 waves of data collection. ^b^Reasons for incomplete variables include but are not limited to cast, wheelchair, or other injury; wearing a dress, braids, or other hairstyle that impeded height measurement; and refusal to remove socks, left early, and unable to finish measures.

Table 1 shows the child demographic and adiposity characteristics between the sample without preintervention and postintervention blood values (2442 children) and subsamples A and B. Of the 695 children in subsample A, 307 (44.17%) were male, their mean (SE) age was 9.28 (0.04) years, 480 (69.02%) were Hispanic, and 452 (65.03%) were eligible for FRL. Children in subsample A were significantly more likely to be Hispanic, less likely to be White, had higher BMI and BMI percentiles, and had higher overweight and obesity prevalence compared with children without preintervention and postintervention blood draws. Children in subsample B also had higher BMI and BMI percentiles and had higher overweight and obesity prevalence compared with children without preintervention and postintervention blood draws; however, there were no differences in race and ethnicity between these 2 samples. In addition, there was a greater proportion of children in the intervention group compared with the control group who participated in the blood draw vs those who did not (354 children [51.0%] vs 1059 children [43.4%]).

**Table 1.** Child Demographic and Adiposity Characteristics Between Sample Without Preintervention and Postintervention Blood Values, and Subsample A and Subsample B

Characteristics	Children, No. (%)			*P* value^c^	*P* value^d^
	Sample without blood draw	Subsample A (n = 16 schools)^a^	Subsample B (n = 10 schools)^b^		
Sample size	2442	695	457	NA	NA
Age, mean (SE), y	9.22 (0.92)	9.28 (0.04)	9.33 (0.04)	.17	.18
Sex					
Female	1271 (52.05)	388 (55.83)	254 (55.58)	.18	.17
Male	1171 (47.95)	307 (44.17)	203 (44.42)		
Race and ethnicity					
Black	220 (9.00)	71 (10.28)	44 (9.62)	.35	.86
Hispanic	1583 (64.82)	480 (69.02)	297 (64.99)	.05	.72
Native American, Asian, and Pacific Islander	122 (5.00)	42 (6.02)	29 (6.35)	.22	.30
White	517 (21.17)	102 (14.68)	87 (19.04)	<.001	.78
Eligible for free and reduced-price lunch	1662 (68.06)	452 (65.03)	296 (64.77)	.16	.67
BMI, mean (SE)^e^	19.86 (4.50)	20.69 (0.18)	20.77 (0.22)	.04	<.001
BMI percentile, mean (SE)	69.42 (29.23)	74.38 (1.06)	74.62 (1.30)	<.001	<.001
BMI *z* score, mean (SE)	0.74 (1.13)	0.95 (0.04)	0.95 (0.05)	.12	.13
BMI percentile overweight or obese	1083 (44.35)	355 (51.08)	244 (53.39)	<.001	<.001
Body fat percentage, mean (SE)	25.62 (8.85)	27.11 (0.35)	27.16 (0.42)	.32	.33
Proportion of intervention participants	1059 (43.40)	354 (51.00)	236 (51.60)	<.001	<.001

Abbreviations: BMI, body mass index; NA, not applicable.

^a^ Data are complete case analyses for participants with complete fasting glucose at baseline and postintervention.

^b^ Data are complete case analyses for participants with complete hemoglobin A_1c_ at baseline and postintervention.

^c^ χ^2^ tests (for categorical variables) and independent *t* tests (for continuous variables) were run to test differences in sample without blood draws vs subsample A.

^d^ χ^2^ tests (for categorical variables) and independent *t* tests (for continuous variables) were run to test differences in sample without blood draws vs subsample B.

^e^ BMI is calculated as weight in kilograms divided by height in meters squared.

Table 2 shows child demographic and adiposity characteristics between intervention and control groups in subsamples A and B. There were no differences in age, sex, race and ethnicity, FRL eligibility, BMI parameters, or BMI status between intervention and control children in subsample A or B.

**Table 2.** Child Demographic and Adiposity Characteristics Between Subsamples A and B Intervention and Control Groups

Characteristics	Subsample A (n = 16 schools)^a^			Subsample B (n = 10 schools)^b^		
	Mean (SE)		*P* value^c^	Mean (SE)		*P* value^c^
	Intervention (n = 8 schools)	Control (n = 8 schools)		Intervention (n = 5 schools)	Control (n = 5 schools)	
Sample size	358	337	NA	236	221	NA
Age, y	9.27 (0.05)	9.29 (0.05)	.68	9.35 (0.05)	9.31 (0.06)	.63
Sex, No. (%)						
Female	193 (53.91)	195 (57.86)	.19	134 (56.78)	120 (54.30)	.59
Male	165 (47.55)	142 (42.51)		102 (43.2)	101 (45.70)	
Race and ethnicity, No. (%)						
Black	35 (9.77)	36 (10.78)	.85	22 (9.32)	22 (9.95)	.91
Hispanic	248 (69.27)	232 (68.84)	.78	151 (63.98)	146 (66.06)	.61
Native American, Asian, or Pacific Islander	21 (5.87)	21 (6.23)	.26	14 (5.93)	15 (6.79)	.99
White	54 (15.08)	48 (14.24)	.54	47 (19.92)	40 (18.10)	.48
Eligible for free and reduced-price lunch	226 (63.13)	226 (67.06)	.46	53 (64.83)	143 (64.71)	.93
BMI^d^	20.59 (0.25)	20.79 (0.26)	.58	20.78 (0.31)	20.77 (0.31)	.98
BMI percentile	73.53 (28.53)	75.27 (26.53)	.41	73.99 (1.86)	75.29 (1.81)	.62
BMI *z* score	0.98 (0.06)	0.92 (0.06)	.58	0.97 (0.07)	0.94 (0.07)	.74
BMI percentile overweight or obese, No. (%)	178 (49.72)	177 (52.52)	.66	122 (51.69)	122 (55.20)	.45
Body fat percentage	27.01 (8.88)	27.21 (0.50)	.78	27.19 (0.59)	27.12 (0.60)	.94

Abbreviations: BMI, body mass index; NA, not applicable.

^a^ Data are complete case analyses for subjects with complete fasting glucose at baseline and postintervention.

^b^ Data are complete case analyses for subjects with complete hemoglobin A_1c_ at baseline and postintervention.

^c^ χ^2^ (for categorical variables) and independent *t* tests (for continuous) were run to test differences between intervention and control groups in subsamples A and B.

^d^ BMI is calculated as weight in kilograms divided by height in meters squared.

Complete case analyses of intervention effects on metabolic parameters are shown in Table 3. Compared with children in the control schools, children in the Texas Sprouts intervention had a 0.02% reduction in mean HbA_1c_ (95% CI, 0.03%-0.14%; *P* = .005) and a 6.40 mg/dL reduction in mean LDL cholesterol (95% CI, 3.82-8.97 mg/dL; *P* = .048). There were no intervention effects on glucose, insulin, HOMA-IR, or other lipid parameters.

**Table 3.** Texas Sprouts Intervention Effects on Metabolic Outcomes

Outcomes^a^	Intervention (n = 8 schools)			Control (n = 8 schools)			Differences in changes, mean (SE) [95% CI]	*P *value, intervention effect^b^
	Children, No.	Mean (SE)		Children, No.	Mean (SE)			
		Preintervention	Change		Preintervention	Change		
Fasting glucose, mg/dL	358	90.84 (0.29)	6.53 (0.29)	337	93.70 (0.24)	2.34 (0.18)	4.22 (1.22) [1.60 to 6.84]	.13
Fasting insulin, μU/mL	348	17.02 (0.09)	0.84 (0.14)	335	15.81 (0.15)	1.26 (0.08)	0.61 (0.80) [−1.16 to 2.37]	.46
Homeostatic model assessment of insulin resistance	348	3.63 (0.02)	0.68 (0.03)	335	3.50 (0.03)	0.57 (0.02)	0.39 (0.18) [−0.001 to 0.77]	.84
Total cholesterol, mg/dL	347	154.57 (0.17)	−7.35 (0.19)	334	151.04 (0.15)	−3.23 (0.30)	−5.72 (−1.24) [−8.39 to −3.04]	.12
Low-density lipoprotein cholesterol, mg/dL	347	88.00 (0.07)	−8.43 (0.22)	334	84.46 (0.15)	−3.53 (0.26)	−6.40 (−1.20) [−8.97 to −3.82]	.048
Triglycerides, mg/dL	347	91.49 (0.42)	5.92 (0.54)	334	89.26 (0.48)	1.42 (0.41)	1.52 (2.54) [−15.42 to 6.47]	.39
HDL cholesterol, mg/dL	347	48.07 (0.14)	0.28 (0.07)	334	48.13 (0.08)	0.59 (0.07)	0.43 (0.34) [−0.30 to 1.17]	.74
Non-HDL cholesterol, mg/dL	347	105.89 (0.06)	−7.62 (0.18)	334	102.95 (0.22)	−3.79 (0.24)	−6.13 (−1.05) [−8.38 to −3.89]	.08
Hemoglobin A_1c_, %^c^	236	5.27 (0.01)	−0.03 (0.02)	221	5.22 (0.01)	0.06 (0.02)	0.02 (0.01) [0.03 to 0.14]	.005

Abbreviation: HDL, high-density lipoprotein.

SI conversion factors: To convert HDL cholesterol to millimoles per liter, multiply by 0.0259; hemoglobin A_1c_ to percentage of total hemoglobin, multiply by 0.01; low-density lipoprotein cholesterol to millimoles per liter, multiply by 0.0259; glucose to millimoles per liter, multiply by 0.0555; insulin to picomoles per liter, multiply by 6.945; total cholesterol to millimoles per liter, multiply by 0.0259; triglycerides to millimoles per liter, multiply by 0.0259.

^a^ Complete case analyses include all children who had blood glucose values available before and after intervention.

^b^ Generalized linear mixed models with the identity link for continuous variables were used to test differences in metabolic parameters between the intervention and the control estimates, with schools as clusters fixed for their intervention effect and children nested within schools.

^c^ Children at 5 intervention schools and 5 control schools were analyzed for hemoglobin A_1c_.

## Discussion

Schools provide ideal settings to reach large amounts of children, and most states mandate that elementary schools implement programs to enhance nutrition and child health.^26^ School gardening programs have consistently been shown to increase FV consumption.^27,28^ To our knowledge, Texas Sprouts was the first cluster RCT to show that a school-based gardening, nutrition, and cooking intervention can improve glucose control and lower LDL cholesterol in elementary schoolchildren.

Although numerous nutrition intervention studies^29,30^ conducted in clinical or community settings have resulted in improvements in metabolic outcomes in children, such as improved glucose control and reductions in lipids, these were not delivered in school settings or used a cluster RCT design. Most school-based interventions that have examined the effect on metabolic outcomes have been multicomponent and included physical activity programming. A cluster RCT^18^ multicomponent lifestyle intervention (that included nutrition, physical activity, behavioral therapy, and social marketing) resulted in reductions in insulin levels compared with control. Another multicomponent school-based cluster RCT, called Bienestar Health Program,^16^ which included nutrition and physical activity education, a family program, a school cafeteria program and after-school health club, resulted in significant reductions in FPG levels compared with control schools over the course of 1 school year. The current findings show that a cluster RCT focused on solely on nutrition, gardening, and cooking components can improve glucose control and reduce LDL cholesterol.

There are several mechanisms to consider in the current study. As previously reported,^15^ the Texas Sprouts intervention resulted in significant increases in daily vegetable intake, approximately one-half a serving a day, compared with control, as measured with a dietary screener data. In addition, Texas Sprouts vs control resulted in a significant increase in Healthy Eating Index 2015 total vegetable scores using 24-hour diet recalls collected in a subsample of children.^31^ There was also a nonsignificant increase in dietary fiber intake in the intervention group compared with control group (0.7 g per day vs no change), using the dietary recall subsample.^31^ Dietary fiber classified into water-soluble fiber and water-insoluble fiber has been considered a leading dietary factor in the prevention and treatment of metabolic diseases, especially lowering LDL cholesterol, in children and adults for over 4 decades.^32,33^ The cholesterol-lowering effect of water-soluble fiber may be a combination of increased fecal bile salts excretion and reduced glycemic response of food, whereas the insoluble fiber may contribute to increased satiety levels.^34^ Dietary fiber has also been shown to improve glucose control and improve microbiota diversity in the gut.^35^ All of these mechanisms may explain how increases in vegetable and fiber intake can improve glucose control and lower LDL cholesterol.

Children in the Texas Sprouts intervention schools and control schools both had an increase in added sugar intake but to a lesser extent within the Texas Sprouts group (0.3 vs 2.6 g per day).^31^ Added sugar intake has been implicated in increased risk for obesity, dyslipidemia, cardiovascular disease, and type 2 diabetes. Experimental studies^36^ have shown that added sugar intake between 8% and 30% of total energy intake has been linked to increased glucose, insulin, and insulin resistance. The effects of added sugar intake on LDL cholesterol have been variable. Some experimental studies show that large doses of sugar have been linked to increases in LDL cholesterol,^37,38^ whereas others have not demonstrated such increases.^39^ Another potential mechanism is that added sugar intake increases energy consumption and can lead to weight gain and increased adiposity. However, the current intervention did not significantly reduce energy intake or lower obesity or adiposity levels, and the changes in dietary intake were independent of changes in energy intake.

The Dietary Reference Intakes for fiber recommend that children aged 8 to 11 years consume 20 g per day^40^; however, 95% of US children fall short of meeting this recommendation, with the average child consuming 12 to 14 g per day.^41^ Although Texas Sprouts resulted in an increase in consumption of dietary fiber, children were consuming less than 14 g per day of dietary fiber after the intervention, which is significantly less than recommended amounts.^31^ The Dietary Guidelines for Americans recommend 2.5-cup equivalents of vegetables per day for children aged 9 to 13 years, and National Health and Nutrition Examination Survey data indicate that more than 90% of US children aged 2 to 18 years fail to meet these recommendations.^9^ The Dietary Guidelines for Americans also recommend that less than 10% of calories a day should come from added sugar intake. Our previous findings^31^ showed that Texas Sprouts children got closer to meeting those added sugar recommendations compared with the control group (10.4% vs 11.0%). The current findings suggest that small increases in dietary fiber and vegetable intake and reductions in added sugar intake may have combined effects on lowering LDL cholesterol and improving glucose control.

Most nutrition interventions delivered in schools have only examined the effects of the intervention on changes in dietary intake and anthropometrics, such as BMI parameters, with moderate success in improving diet and little to no success at reducing obesity. The current intervention resulted in reductions in LDL cholesterol and improvements in HbA_1c_, independent of changes in body composition. Similarly, other multicomponent school-based programs that have resulted in improvements in glucose levels, insulin levels, or both, even though they had no effect on BMI.^16,42^ Numerous school-based interventions have failed to move the dial on reducing obesity, yet still have merit because they were successful at reducing cardiometabolic disease risks. There is a need for more school-based cluster RCTs to assess the effects of nutrition interventions on metabolic outcomes, independent of weight or body composition change in youth.

It is important to note that there were no intervention effects on glucose, insulin, HOMA-IR, or other lipid parameters, such as triglycerides, total cholesterol, non-HDL cholesterol, and HDL cholesterol. The study was powered on the primary outcome of FPG, which did not significantly change between groups, whereas the other metabolic parameters were secondary outcomes. One possible explanation for the null effects on glucose and insulin is that collection of glucose and insulin at a single time point may not have been representative of that child’s usual blood glucose and insulin secretion response. Collecting multiple glucose and insulin samples from a frequently sampled IV glucose tolerance test, or during an oral glucose test or meal challenge test, are more accurate ways of assessing glucose control than a single-time-point collection,^43^ but those tests are expensive, time-consuming, and often not an option for in-school testing. HbA_1c_ also uses a onetime collection, can be collected in school or community settings, and reflects average glycemia over approximately 3 months. Another limitation is that this study did not collect data on pubertal status, which could give insight on the theories behind associations of age and glucose control.

### Limitations

There are several other limitations to mention. The first limitation is that multiple imputations could not be run on the missing data of the survey data with the blood variables, because too many variables were included to make the analysis congenial and the imputation models would not converge with the blood variables, so complete case analyses was run. The subsample of children who participated in the optional blood draw had higher BMI, BMI percentiles, and overweight and obesity prevalence compared with children who did not participate in the blood draw. One explanation for this is that parents of heavier children may have been more concerned about their child’s health and opted to receive the free diabetes screening, which is how we marketed this to the families. In addition, the individuals in the subsample with blood draws were more likely to be Hispanic and less likely to be White, and Hispanic individuals in this population may have less access to health care, which could have encouraged them to sign up for the free diabetes screening test. Regardless, it appears that the subsample with blood draws represented a higher risk group; thus, the results cannot be generalized to healthier pediatric populations. However, these results highlight that this intervention could be effective at reducing metabolic disease risk in a high-risk subset of children. Another limitation is that the HbA_1c_ values were only measured in waves 2 and 3 and, therefore, included only 10 schools (5 intervention and 5 control schools). As mentioned already, HbA_1c_ was added on the basis of the suggestions from the data safety monitoring board on the higher-than-expected FPG values seen in wave 1. In addition, children in the intervention group compared with control were also more likely to participate in the blood draws. It is possible that the garden that was built the prior spring and the excitement of being in the garden program the upcoming school year encouraged them to participate in the blood draw. Another limitation is that the improvement in HbA_1c_ was rather small, albeit significant, and may not be clinically relevant. However, health care professionals believe that even small reductions in HbA_1c_ levels reflect clinical improvements in glucose control.^44^ Another limitation is that the schools selected were a majority Hispanic and low income, and the results of this school-based intervention may not be generalizable to other populations. However, given that low-income and Hispanic children are at higher risk of obesity and related metabolic diseases, having cluster RCTs targeting health improvements in schools serving children from low-income and minoritized groups are warranted. Another limitation is that this study was only 9 months long and that no follow-up postintervention data were collected. Furthermore, we provided the educators who taught the Texas Sprouts lessons; thus, scaling and sustaining this program in a school setting without external educators might be challenging. However, there are national efforts to provide training, curriculum, and resources to schoolteachers across the nation to help them develop and sustain school gardening programs.^45^

## Conclusions

In conclusion, the present RCT showed that a school-based gardening, nutrition, and cooking intervention resulted in a small, albeit significant, improvement in glycemic control and a reduction in LDL cholesterol in predominately low-income and racially diverse elementary schoolchildren. Given that there is a critical need to reduce obesity-related metabolic disease in children, especially in low-income and Hispanic populations, this intervention has the potential to be implemented and scaled across the US. Teaching garden-based nutrition education programs allows public schools to meet nutrition education state mandates, while potentially reaching nearly 24 million kindergarten through fifth grade children in the US. School-based gardening programs improve dietary intake, academic performance, and reduce metabolic diseases in even the most high-risk minority pediatric populations. These findings provide direct evidence to help encourage policy makers, administrators, and school district personnel to adopt and/or support garden-based learning into elementary schools.

## Notes

Supplement 1.
Trial Protocol

Supplement 2.
Data Sharing Statement
