# Supplementary material for: Effects of a School-Based Nutrition, Gardening, and Cooking Intervention on Metabolic Parameters in High-risk Youth: A Secondary Analysis of a Cluster Randomized Clinical Trial
Source: JAMA Netw Open. 2023 Jan 10;6(1):e2250375. doi: 10.1001/jamanetworkopen.2022.50375 (PMC9856961; doi:10.1001/jamanetworkopen.2022.50375)
Supplement: Supplement 1. — Trial Protocol [file jamanetwopen-e2250375-s001.pdf]

Research Proposal

1. **Title:** TX Sprouts

2. **Principal Investigator:** Jaimie Davis PhD, RD, Assistant Professor, Department of Nutritional Sciences, UT-Austin

   Co-Investigator & Study Pediatrician: Steven Pont, MD; Pediatrician and Director of Childhood Obesity Center, Dell Children's Hospital

   Key Personnel: Katharine Nikah, MPH, Project Coordinator, Department of Nutritional Sciences, UT-Austin

3. **Purpose:**

School gardening programs offer affordable and accessible fruits and vegetables (FV), teach children how to grow, cook, and enjoy FV, and show promising effects on reducing obesity and related diseases in low-income Hispanics. Lack of access to healthy and affordable food is a significant factor contributing to elevated rates of obesity and metabolic risk factors among low-income Hispanics. There is a growing national and grassroots movement to support gardening efforts to make healthy foods more affordable and accessible and to make intake more sustainable for low-income families. However, few experimental studies have evaluated the impact of garden-based interventions on obesity and related metabolic disorders.

The overall goal of this project is to test the effects of a large school-based gardening, nutrition, and cooking randomized control trial (called "TX Sprouts") on dietary intake, dietary-related behaviors, obesity, and related metabolic disorders in low-income Hispanic youth and their families in Central Texas. Elementary schools will be randomized to either: 1) TX Sprouts intervention or 2) Control (delayed intervention). At each intervention school, we will build edible gardens; form and train Garden Leadership Coalitions (GLCs); teach 20 TX Sprouts in-school lessons to the students; and teach nine family-based TX Sprouts lessons throughout school year.

After the intervention year, we will provide a series of training workshops and resources to the GLCs and schoolteachers to sustain the TX Sprouts program in subsequent years. We will measure the sustainability employed by each school by process logs/surveys, structured interviews, surveys and school observations. The findings could provide a successful, sustainable model for garden-based school programs that decrease the obesity and metabolic diseases impacting Hispanic populations.

All participating school will get the TX Sprouts program regardless, because it is taught during school hours. This research study is to be allowed to conduct evaluation measures. The related consent forms pertain to the research/evaluation portion of this proposal.

<u>Aim 1:</u> To test the effect of the Sprouts intervention on the following changes in health outcomes for the child: a) FV intake; b) dietary psychosocial variables; c) anthropometric parameters (e.g., BMI, WC, percent body fat); d) metabolic risk factors (e.g., blood pressure, metabolic syndrome, fasting glucose, insulin, and lipids); and e) home environment (e.g., availability and accessibility of FV).

Hypothesis 1: Sprouts intervention students compared to control students will have, as primary outcomes: increased vegetable intake and reduced BMI z-scores, WC, fasting glucose, and insulin; and, as secondary outcomes: increased fruit and fiber intake and reduced added sugar intake, improved dietary psychosocial variables (i.e., preference and motivation, self-efficacy for FV), and reduced body fat and other metabolic risk factors.

<u>Aim 2:</u> To test the effects of the TX Sprouts intervention on changes in dietary intake and related dietary psychosocial variables of the parents.

Hypothesis 2: TX Sprouts intervention parents compared to control parents will have increased FV intake, improved psychosocial variables linked to FV intake, and improved access and availability of FV in the home.

Exploratory Aim: To identify specific strategies/components of the TX Sprouts intervention that were implemented and sustained at the completion of the intervention, which will provide valuable information on how to disseminate and sustain long-term garden programs in schools.

4. Procedures:

TX Sprouts Program:

Sixteen elementary schools (~4,800 3rd-5th grade students) from four independent school districts have been identified with: a) >50% Hispanic children; b) >50% participation in the free/reduced school lunch program; c) located within 60 miles of the University of Texas at Austin campus; d) high prevalence of pediatric obesity; and e) interest in a school garden/nutrition program. Elementary schools will be randomized into either: 1) TX Sprouts Intervention or 2) Control (delayed intervention). At each intervention school, we will build edible gardens; form and train Garden Leadership Coalitions (GLCs); teach 20 TX Sprouts in-school lessons to the students; and teach nine family-based TX Sprouts lessons throughout school year. The control schools will serve as the comparison group and will receive a delayed intervention the next year.

Garden Leadership Coalition (GLC): We will establish GLCs at each of the schools during the intervention phase, which will facilitate some of the school-level components of the intervention. These GLCs will be comprised of interested teachers, school staff, senior citizens, parents, and children, but these groups will be different and unique at each school site. . Given that the CATCH program is currently being implemented in all participating school districts as the state-mandated coordinated school health programs, we will enlist the CATCH Champions and Wellness Committees at each school to be a part of the GLC. Key personnel (Drs. Davis, Warren, Evans, and Hoelscher) have considerable experience with coalition building in schools. We will employ a GLC Coordinator to help form and organize GLCs at each school site and serve as the liaison between the GLCs and the Sustainable Food Center (SFC), a nationally recognized Austin-based non-profit organization that promotes gardening and health eating in a culturally relevant manner. SFC has developed and currently teaches School Garden Leadership Trainings across the Austin area. SFC will host a Garden Leadership Training workshop during the intervention phase and will then teach a series of three workshops to the GLCs in the subsequent year. Currently, SFC offers several workshops, including: Basic Organic Gardening classes (which includes information on planting fruit trees, rain water collection, fence building, and gardening in pots/containers), Farm Direct classes (which connect local growers with schools), and La Cocina Alegre (a series of family cooking classes using fresh produce). The GLC Coordinator will work with the GLCs to select and provide these workshops to the schools; the cost of the workshops will be covered by this grant. The grant will also pay for an AgriLife Extension Coordinator to help provide local Master Gardener volunteers to work with the GLCs to help maintain the gardens and sustain the gardening program. As part of the Extension Master Gardener program, all volunteers undergo a 3-month training program and then must complete 50 hours of volunteer work the first year and 30 hours of volunteer work every year after, so this partnership is ideal for their program as well as the school sites. The GLC coordinator, the Extension Coordinator, and the Master Gardener volunteers will continually help connect the GLCs to local stakeholders, including local agricultural producers, community gardens, food pantries, food banks, and senior citizen centers. GLCs will be encouraged to meet before or after school twice a month in the beginning and at least monthly for the remaining years. The GLCs will be responsible for holding design-build meetings and for planning and implementing the Garden Kick-Off and Harvest Celebration events. GLCs will develop an action plan to engage school maintenance staff, teachers, and other community members to get involved in the program as well as reach out to garden sponsors and local foundations to fundraise in subsequent years. Other potential strategies that the GLC might employ include: hosting family plant and harvest days throughout

the year, hosting community engagement demonstrations, (i.e., FV stands, farmer's markets, and recipe demonstrations), and contacting local media outlets to do stories on garden activities/program. Of note, every GLC will likely implement and adapt different strategies from a menu of evidence-based options, which is why we plan to develop a list of these options, and measure these strategies in both intervention and sustainability years as done in the CATCH Travis County study.[1] Forming these GLCs will empower the community members to take ownership in this program and continue to maintain the gardens and sustain the program after the grant cycle is over.

Edible Gardens: We have support from school districts and the individual schools to build gardens at each of the school sites. Raised beds will be used at all garden sites, as these are easy to build, make garden access easy for children of all developmental levels, ensure that soils are free of heavy metals/contaminants, and reduce weeds in the garden area. We have support from school districts and the individual schools to build gardens at each of the school sites. Raised beds will be used at all garden sites, as these are easy to build, make garden access easy for children of all developmental levels, ensure that soils are free of heavy metals/contaminants, and reduce weeds in the garden area. The beds will be designed by the GLCs at each school during a "design-build" session with input from the maintenance staff and principals as well as assistance from Texas AgriLife Extension staff and the SFC. Supplies will be purchased from this grant, with the local horticulturist's guidance, and delivered to the appropriate garden site. A garden kick-off event at each garden site will engage students, teachers, practitioners, parents, and community volunteers in a garden build. Each school site will get the materials and supplies needed for garden upkeep (e.g. rakes, hoses, etc.) and for teaching the lessons, (e.g., tables, chairs/benches, cooking grill, portable hand-washing sink, pots/pans, etc.). A big media/kick-off event will be hosted at each school after each garden is built. In addition, the Extension Volunteer Coordinator will place and oversee Master Gardener volunteers who live within each of these communities to assist with the build and upkeep of the garden in the intervention year, as well as subsequent years

Curriculum: We will use an adapted version of the LA Sprouts curriculum. LA Sprouts includes 12 lessons, each approximately 90 minutes in length), and we will break it down to 20 lessons, each 30-45 minutes in length taught during intervention year of this project. The following nutrition topics are included in the curriculum: a) healthy cooking/preparation of FV (i.e., low in sugar and fat); b) FV preservation; c) eating locally produced food; d) low-sugar beverages made with fresh FV; e) health benefits of FV; f) how to eat healthfully in food desert neighborhoods; and g) food equity and community service. The curriculum also covers a broad range of horticultural and environmental education topics including: science process skills, observation, taking measurements, and problem solving through both group and individual learning experiences. The curriculum is culturally tailored to Hispanics, including culturally appropriate recipes, content, and activities. In addition, Extension materials and the SFC programs are available in Spanish and are culturally appropriate. The curriculum is mapped on Texas Essential Knowledge and Skills (TEKS) standards for science, math, language arts, health, and social studies.

For the parent lessons, we will use the developed and tested LA Sprouts family curriculum that parallels the nutrition and gardening topics/activities taught to the children. The LA Sprouts family curriculum includes twelve 90-minute lessons, which will be divided into nine 60-minute lessons for the proposed project. The parent curriculum is available in both English and Spanish and has been culturally adapted and evaluated. The curriculum has a strong emphasis on the cooking components and focuses on growing and cooking foods that are culturally appropriate. Often, the children are given the opportunity to teach their parents how to cook healthy meals with FV. This model empowers the child to be the champion for healthy changes in the family. Some of the more popular activities in this curriculum include: family cooking competitions, recipe development and sharing, and fall/spring harvest celebrations.

School Lessons: All 3rd-5th-grade students in the participating schools will receive the curriculum as part of their normal school day. We will hire bilingual nutrition and garden educators who have experience teaching nutrition/ gardening programs to teach the TX Sprouts curriculum in the Intervention year. All educators will undergo a weeklong training course led by the research and Extension staff that will cover all material/activities taught during each lesson, structure and flow of each class, cooking and preparation

skills needed to make recipes in curriculum, classroom preparation/clean up procedures, logistical issues with teaching in garden, how to complete process logs, and any issues with supervising student volunteers. The schoolteachers will "shadow" our educators during these lessons the first year. Twenty TX Sprouts lessons (each 30-45 minutes in length) will be taught by our educators separately to each 3rd-5th grade classroom throughout the school year as part of their normal school day. Before the program is implemented, we will work with stakeholders in each school district to decide exactly where and when these lessons can be taught and incorporated into normal school days.

Family Lessons at Schools: The bilingual garden/nutrition educators will also teach monthly TX Sprouts lessons throughout the school year for the families of participants at each of the schools, for a total of nine lessons. Each lesson will be taught by a bilingual gardening/nutrition educator and will consist of a 60-minute lesson that parallels the topics taught during the student lessons. We will incentivize the parents to attend the lessons by offering free meals, produce, groceries, and free babysitting for younger siblings.

Delayed Intervention to Control Schools: The control schools will receive a delayed intervention (identical intervention as described above) in the year after the post-testing for that wave. Baseline and post-intervention measurements will occur in the control parents and students within the same time period as the intervention schools. Every control school will receive a garden, identical in size and structure to the intervention schools. We will teach school lessons as well as offer monthly family lessons at the schools. We have used the delayed intervention control group in the past with success, and it provides incentives for families and schools to stay involved in the study as in the control group

TX Sprouts Research Evaluation:

Evaluation measures will be collected at baseline and end of the school year in both treatments (intervention and control schools). Measurements will occur during the first four weeks of school in the fall and the last four weeks of school in the spring. Most measures on children will be collected at the schools during normal school hours. The fasting blood draws will occur first thing in the morning before school starts. The following evaluation measures will be obtained from students at baseline and post-intervention:

- Measured height, weight, BMI parameters, waist circumference (WC), body composition (via bioimpedance); carotenoid scan of skin
- Blood pressure via child-specific automated cuff;
- Subsample of students will complete three 24-hour diet recalls by telephone;
- Child survey assessing dietary intake via food screener and related determinants of dietary behaviors (e.g., preference/motivation/self-efficacy to eat FV);
- Program lessons may also be audio/visual recorded;
- Voluntary fasting blood sample to assess glucose, insulin, and lipid parameters;
- Accelerometers to measure physical activity;
- Time on Task observations

Parent questionnaires will be handed out or mailed to the homes after consent is obtained at baseline and post-intervention. Questionnaires are translated into Spanish, culturally appropriate, and take ~60 minutes to complete. We will encourage parents to complete the questionnaire before the first family lesson and return it via mail (pre-paid postage will be provided). However, parents will also be able to complete the questionnaire at the first and last lessons. Parent surveys will evaluate the following: food security via USDA validated questions, confidence cooking, dietary intake via validated screener, reported height and weight, medical history, basic demographics, child feeding practices and food home environment from CEBQ and TGEG, and preferences adapted from Domel et al. We will also attempt to measure height and weight on a subsample of parents, which will occur at the first and last parent lesson. Parent workshops may also be audio/visually recorded.

After the intervention year, we will conduct a series of structured interviews (30 minutes or less per interview) as well as surveys, with participating school stakeholders (i.e., principals, teachers,

administration staff) and members of the GLCs to measure sustainability approaches employed at each school. We will audio recorded these structured interviews. We will provide a sustainability consent form for participants to sign prior to agreeing to the interview. We will also conduct in-school observations to document sustainability measures around the TX Sprouts program.

a. ***Location***

We have partnered with four school districts for this project (see letters of support from superintendents and principals in these districts in Appendix A) Elgin ISD, Manor ISD, Georgetown ISD, and Del Valle ISD. These districts were selected because of their: 1) high proportion of Hispanic children (>50%); 2) high proportion of children participating in the free and reduced lunch (FRL) program (>50%); 3) high proportion of overweight or obese children; 4) location within 60 miles of UT-Austin campus; and 5) interest and support in a garden/nutrition school program. There are 18 schools within these school districts that meet the inclusion criteria. Sixteen out of the 18 schools will be randomly selected from this sampling frame to allocate to the intervention or control arms and the two schools remaining will be used for replacement if a school declines to participate.

Data collection and related study activities will occur at these schools within each school district.

b. ***Resources***

External funds from the National Institutes of Health (NIH) will support this project. Notice of award is pending, but this grant received a fundable score and funding is highly likely. Earliest possible funding start date would be April 1st, 2015. Of note, IRB approval is needed for Just-In-Time NIH request.

c. ***Study Timeline***

The intervention will run in a series of three waves across four academic school years in Years 2-5 (see **Table 1**). Waves 1 and 2 (**W1** and **W2)** will each include six schools (3 intervention (INT) and 3 control (CON) schools), and Wave (**W3**) will consist of four schools (2 INT and 2 CON).

Table 1: General design and timeline of study

Year	Fall	Spring	Summer
1	-Obtain UT-Austin and school district IRB approvals; Hold stakeholders meetings to plan implementation of Sprouts curriculum at each of the schools; Set up database; and Hire and train staff; Randomly allocate schools into INT/CON and into waves; Adapt and modify curriculum.		-Build **W1-INT** gardens
2	-**W1** baseline measurements<br>-Implement **W1-INT** Sprouts program	-Complete **W1-INT** Sprouts<br>-**W1** post-intervention measurements	-Build **W1-CON** gardens<br>-Build **W2-INT** gardens
3	-Host train the trainer workshops for **W1-INT**<br>-Implement **W1-CON** Sprouts program<br>-**W2** baseline measurements<br>-Implement **W2-INT** Sprouts program	-Complete **W1**-CON Sprouts program<br>-Complete **W2**-INT Sprouts program<br>-**W2** post-intervention measurements<br>-Collect sustainability data in **W1-INT**	-Build **W2-CON** gardens - Build **W3-INT** gardens
4	-Host train the trainer workshops for **W2-INT**<br>-Implement **W2-CON** Sprouts program<br>-**W3** baseline measurements<br>-Implement **W3-INT** Sprouts program	-Complete **W2-CON** Sprouts program<br>-Complete **W3** Sprouts program<br>-**W3** post-intervention measurements<br>-Collect sustainability data in **W1-CON**<br>-Collect sustainability data in **W2-INT**	-Build **W3-CON** gardens
5	-Implement **W3-CON** Sprouts program; Host train the trainer workshops for **W3**; Collect sustainability data in **W3-INT;** Host the Results Dissemination Workshop; Data entry, cleaning, and analyses; Manuscript and subsequent grant preparation;		

Of note: *Consenting and measurements in the fall will occur the first 2 weeks of school, and measurements in the spring will occur during the last 2 weeks of school. Data entry, cleaning, and analyses and manuscript preparation will be done continuously in Years 2-5. INT=Intervention schools; CON=Control schools.*

5. **Measures**

Anthropometrics, Body Fat, and Blood Pressure: For both children and parents, weight will be measured using a SECA (model 869) digital scale to the nearest 0.1 kg, and height will be measured using a wall-mounted stadiometer to the nearest 0.1 cm. BMI (kg/m^2) and BMI percentiles for CDC age- and gender-specific values will be determined using EpiInfo 2005, (CDC, Atlanta, GA). Waist circumferences (WC) will be measured and recorded to the nearest 0.1 cm. Body composition will be assessed using Bioelectrical Impedance with the Tanita Body Fat Analyzer (model TBF 300). Blood pressure will be obtained in duplicate with an automatic cuff according to recommendations of the American Heart Association.[2] A skin carotenoid scan will be completed of the finger, which will take approximately 20 seconds. This is a noninvasive device that measures carotenoids in the skin to assess fruit and vegetable consumption. The device uses a technique called reflectance spectroscopy, which involves shining a light on the skin of one finger to measure carotenoids in the skin which gives us an objective measures of vegetable intake.
Parental height, weight, body fat, WC, and blood pressure will be collected at first and last family workshop (expecting a subsample only). Therefore we will ask self-reported height and weight in questionnaire packets.

Dietary Intake: The Block Food Screener for Ages 2-17, 2007[3] will be used to measure dietary intake on all children. This 41-item screener asks about food eaten "yesterday," and was designed to assess children's intake by food group, with a specific focus on fruit and fruit juices, vegetables, potatoes (including and excluding French fries), whole grains, meat/poultry/fish, dairy, legumes, saturated fat, and "added sugars" (in sweetened cereals, soft drinks, and sweets). This screener was designed for self-administration by children with the assistance of an adult and takes ~10 minutes to complete. The screener was developed and adapted from the Block Kids 2004 Food Frequency Questionnaire (FFQ)[4] and was recently validated against this FFQ in 2,376 primarily Hispanic children (8 y).[5] We will also collect 24-hour diet recalls in a subset of children (n=500, half in the intervention and half in the control group). Trained interviewers will collect three 24-hour recalls using the multiple pass technique[6] by telephone (making sure to obtain 1 weekend and 2 weekdays) within 10 days of each testing visit. Dr. Davis has extensive experience training students/staff to collect and enter FFQs and 24-hour recalls. UT-Austin has a large pool of undergraduate dietetics students who will be trained to assist with collection of these recalls as part of their research credit hours. All recall data will be analyzed using the Nutrition Data System for Research (NDS-R 2014, University of Minnesota). Dietary intake for all parents will be measured using Block Fat/Fruit/Vegetable/Fiber screener, which is a validated 10-item screener that is available in English or Spanish.[7]

Demographic and Dietary Psychosocial Measures: Demographic information including age and ethnicity will be obtained via a questionnaire. To ascertain socioeconomic status, we will ask students whether their family uses a computer at home and whether their mother has her own car,[8] and we will ask parents to report income level. Parents will fill out a brief medical history form for their child and themselves to rule out exclusion criteria mentioned above. Children and parents will complete the Motivation for Fruit and Vegetable Intake, Gardening, and Cooking Scale, which has been adapted from the Treatment Self-Regulation Questionnaire (TSRQ).[9,10] We will measure FV preferences as well as self-efficacy to garden/eat/prepare FV in both the child and parent using existing questionnaires that have been developed and validated with pediatric populations.[11-15] The Child Eating Behavior Questionnaire (CEBQ), which is a validated parent-report instrument to assess obesogenic eating behaviors in children,[16] will also be administered. Physical activity (PA) information will be obtained on the child via a 3-day physical activity recall (3-Day PAR), a semi-structured interview that estimates an individual's time spent in PA, strength, and flexibility activities during the three days prior to the interview. This survey has been validated for PA assessment in children.[17] All parent questionnaires will be available in both English and Spanish, and written at a 3rd grade reading level.

Accelerometers

Children will be asked to wear accelerometers throughout the day during the weeks of data collection. These will be used to record their daily physical activity levels and to have a quantitative measure of how much activity they incur during regular classes, recess, etc.

With regard to wearing accelerometers, children will wear them from the start of the school day until the end of the school day for a 5-day period. From previous projects, it has been seen that children enjoy wearing the accelerometers and are excited to have their step counts recorded. Any discomfort about wearing the accelerometer on a belt around the waist is offset by the excitement factor of wearing one. If a child states they are uncomfortable wearing one, they will not be required to wear an accelerometer.

Time on Task: Children will be observed for time-on-task measures during designated class times by trained project staff. The observers will sit or stand in a corner of the classroom and will not interact with the teacher or the students. The observers will be in the classrooms for about 15-20 minutes at a time and then leave quietly. Observers will write field notes on whether the student is staying on task throughout a portion of a lesson. Students are used to having classroom visitors enter and exit the classroom as part of the regular school day, and thus are used to visitors in the rooms. No observation data will be shared with the students or the teachers to maintain confidentiality of observations. Little to no disruption will occur to the classroom setting as the regular lessons are taught.

Fasting Blood Sample: Certified phlebotomists will perform the fasting blood draws on students first thing in the morning before school starts at the schools at baseline and at the end of the school year. Blood draws will occur on school campus in the school health clinic or another designated sterile area. A total of 48 ml of blood will be drawn at baseline and 9 months later at post-testing. Students with parental consent and assent will participate in the blood draw and receive a cash incentive ($20 per draw). Students who do not assent to participate in the blood draw will still be allowed to participate in the Sprouts program and other evaluation measures. A reminder blood draw letter will be send out for post evaluations. We will also send a normal diabetes test to all blood draw participants who do not have elevated glucose levels. Fasting blood will be drawn to assess glucose, insulin, lipids, and adipocytokines. Blood samples will be spun, aliquotted, frozen, and transported to the UT campus for assaying. Glucose will be assayed using a Yellow Springs Instruments analyzer (Yellow Springs, OH) and insulin using an automated enzyme immunoassay system analyzer (Tosoh Bioscience, Inc. San Francisco, CA). Homeostatic model assessment (HOMA-IR) will be used as a measure of insulin resistance.[18] Total cholesterol, high-density lipoprotein cholesterol (HDL), and triglyceride levels will be measured using the Vitros chemistry DT slides (Ortho Clinical Diagnostics Inc., Rochester, NY); low-density lipoprotein (LDL) will be calculated using the Friedwald formula. Adipocytokines (i.e., leptin, adiponectin, interleukin-6) will be measured using the Millipore Multiplex assay kit. Metabolic syndrome will be calculated using a combination of Cruz et al.[19] and Cook et al.[20] definitions. All children who participated in the fasting blood draw will be mailed a copy of their diabetes screening results and those with elevated glucose levels (in the abnormal or T2D range) will be contacted via telephone by Dr. Pont (Co-I on this study) to explain the test results and be referred immediately to a health clinic for follow-up. However, these children will still be allowed to participate in the study.

Sustainability Measures: During the Sprouts intervention and sustainability year, we will collect pilot data on sustainability strategies being implemented in both the intervention and control schools. Structured interviews and audio-recorded meeting data will be conducted with GLC members to document member information, leadership structure, meeting times/places, how the garden was designed/built, workshops offered/attended, fundraising and media events, types of produce harvested, produce use and distribution plans, community resources leveraged, barriers and future action plans. Structured interviews, surveys and audio-recorded meetings will be conducted with teachers, principals, administration staff, GLCs, and district

directors and staff to evaluate various aspects of the program including: a) use and maintenance of garden; b) successful and unsuccessful components/strategies implemented to sustain program; and c) additional material/resources/training requested and/or needed. We will also conduct observations of schools to assess whether the garden is being maintained and examine what aspects of the program are still being maintained and strategies used to sustain the program (see Appendix for structured interview and focus group consent form).

Data Collection: Some study data will be collected and/or managed using REDCap (Research Electronic Data Capture). REDCap is a secure, web application designed to support data capture for research studies, providing user-friendly web-based case report forms, real-time data entry validation (e.g. for data types and range checks), audit trails and a de-identified data export mechanism to common statistical packages (SPSS, SAS, Stata, R/S-Plus). REDCap also provides a powerful tool for building and managing online surveys. The research team can create and design surveys in a web browser and engage potential respondents using a variety of notification methods. The system was developed by a multi-institutional consortium, which includes University of Texas at Austin and was initiated at Vanderbilt University. The database is hosted at the Population Research Center, which will be used as a central location for data processing and management. The PRC server has been cleared for Category-I data collection by UT's Information Security Office. Network transmissions (data entry, survey submission, web browsing, etc.) in REDCap are protected via Secure Sockets Layer (SSL) encryption. REDCap data collection projects rely on a thorough study-specific data dictionary defined in an iterative self-documenting process by all members of the research team with planning assistance from the PRC. The iterative development and testing process results in a well-planned data collection strategy for individual studies. REDCap provides a secure, web-based application that is flexible enough to be used for a variety of types of research, provide an intuitive interface for users to enter data and have real time validation rules at the time of entry.

Sample Size and Power Analysis: ***For Aim 1:*** We estimated the power of this study for children using pilot data from five primary variables from LA Sprouts: vegetable intake (serv/d), BMI z-scores, waist circumference, fasting glucose (mg/dL) and fasting insulin (μU/ml). We estimated minimum sample size using several effect sizes on the difference between intervention and control group on the change of

Table 2: Minimum number of schools required evaluating the differences in the effect sizes between

			Children (cluster average size of n=127 for diet, waist, BMI z-scores and 60 for glucose/insulin values per school)					Parents (cluster average size of n=64 per school)
			Vegetable Intake	Blood Glucose	BMI Z score	Waist circumference	Insulin	Parents: Vegetable - Intake or Availability
Type I error level			ICC=0.071	ICC=0.001	ICC=0.002	ICC=0.019	ICC=0.554	ICC=0.071
			σ^2=0.08	σ^2=40.86	σ^2=0.05	σ^2=0.005	σ^2=30.41	σ^2=0.08
		effect size	0.500	2.133	-0.065	-0.020	-12.000	0.510
0.05	power	0.8	12	6	4	12	14	12
		0.9	16	8	6	16	20	16
0.01		0.8	18	8	6	18	24	18
		0.9	22	10	8	22	30	24

ICC: intracluster correlation coefficient; σ^2: variance within schools from pilot data

measurements from baseline to end of study. We assumed that a cluster average size of 127 children per school would have pre and post measurements for vegetable intake, BMI z-scores, and waist circumference but only 60 children per school for blood glucose and insulin values. **Table 2** presents the minimum number of schools needed for power of 80% and 90% for the children's primary outcomes using a type I error of 0.05 and 0.01, a two-sided test, and assuming equal allocation between the two arms.[21,22] The variance (σ^2) within schools and the intracluster correlation coefficient (ICC) to estimate the sample size were obtained from the R21 pilot study. Therefore, with six schools and 60 children per school with blood draws, 360 children are needed, and given our preliminary data and attrition rates, we will have enough power to detect the effect size of a decrease in 2.13 mg/dL in fasting glucose. For the children, we will also have enough power to detect an effect size above 0.5 in vegetable (serv/d) intake, a decrease of 0.065 in BMI z-scores, a decrease of at least 0.02 cm in waist circumference, and a decrease of at least 12 μU/ml in fasting insulin. ***For Aim 2***, we estimate the power of this study for the parents using vegetable intake and availability from Castro et al[23] using the same powers and type I error levels as we used for the

children as well as the R21 pilot data for the variance and ICC. Among the parents, we will have enough power to detect an effect size above 0.5 vegetable intake (serv/d) (**Table 2**).

Data Analyses: Summary statistics, graphical analyses, and frequency distributions will be used to describe the data between the intervention and control groups. Data will be transformed if needed. We will handle missing data using two primary approaches: multiple imputation and full information maximum likelihood estimation methods.[24-26] Each method has potential biases and makes assumptions about the underlying basis for the missing data (e.g., missing at random-MAR) and other features of the data that are present. However, we have included enough baseline variables of the participants and at the follow-up visits to make the MAR assumption plausible. Without prior knowledge of the missing data process, we will have to explore the implications of applying different strategies. **For Aim 1**: We will test if the children and parents participating in Sprouts compared to control will have increased changes in average consumption of vegetable servings per day and decreased average changes in BMI z-scores, waist circumference, fasting glucose, and insulin using well-known statistical methods for cluster randomized trials.[21,22] Several analytic strategies will be employed to determine the effect of the intervention on vegetable intake and many of the other primary outcome variables. We plan to conduct a two-stage nested analysis of variance model, which is a special case of the mixed effects linear regression model also known as a repeated measures analysis of variance model. The terms in the model include the true mean for the outcome variable, the fixed effects of the intervention arm, one random cluster effect (school), and the random error term. In this case, the schools (clusters) should be regarded as nested within the intervention arms and the students or parents within the school (clusters). We will model the pattern of covariance among observations as an unstructured variance–covariance matrix because of the small number of repeated assessments. We will also explore interaction effects between intervention group and selected covariates to determine whether the intervention was more effective for some covariates. For example, we will test whether the effectiveness of the intervention varied by age, gender, race/ethnicity, baseline BMI, percent free and reduced lunches, physical activity measures, class attendance, and wave of data collection. These tests will be considered as exploratory analysis and treated as hypothesis generating and interpreted with caution. The analytic approach for the secondary outcome measures (i.e., blood pressure, percent body fat, metabolic syndrome, adipocytokines, other dietary intake variables, and related dietary psychosocial behaviors) will involve a similar use of linear mixed models. Again, school will be modeled as a random effect nested within treatment arm, and treatment arm will be modeled as fixed effects. **For Aim 2:** To test the effects of the Sprouts intervention on changes in vegetable intake and availability and on related dietary behaviors in the parents, we will use similar and well-known statistical methods as described for Aim 1. As exploratory and secondary analyses, we intend to conduct structural equation modeling (SEM) with established mediational (and direct) effects, with and without the cluster effect variables[27,28], to examine how changes in behaviors, as well as program fidelity (attendance, dosage, delivery of intervention) impacts changes in main health outcomes. For the **Exploratory Aim**, we will conduct descriptive statistics on the collected pilot data of the sustainability strategies (qualitative and quantitative data obtained via structured interviews and focus groups) on both intervention and control schools. The unit of analysis will change, as these measurements will be collected on teachers, principals, GLCs, administration staff, district directors and staff. We will obtain pilot data on the differences between intervention and control schools on the following: number of curriculum lessons taught, use and maintenance of garden, successful components/strategies implemented to sustain program, and additional material/resources/training requested, etc. This pilot data will serve to consider future projects to sustain the program and for power and sample size purposes.

6. Participants

a. <u>Target Population</u>

This study targets communities serving primarily low-income Hispanic children, a rapidly growing population in Texas at high risk for obesity and related metabolic disease such as cardiovascular disease and T2D. More culturally appropriate interventions targeting this high-risk population are warranted. The schools from the sampling frame average approximately 300 3rd-5th grade students for a total of 4,800 3rd-

5th grade students and a parent/guardian, as a dyad. We plan to recruit a total of 2,400 students, 1,200 from intervention and control schools respectively. Based on blood draw participation rates in LA Sprouts, we anticipate that 50% of participating children (n=1,200) will also participate in the blood draws at pre-intervention and 40% (n=960 or 60 per school) at post-intervention. We plan to audio-record meetings and conduct structured interviews on approximately 100 school stakeholders and GLC members (20 from each school) to evaluate sustainability.

b. Inclusion/Exclusion

Inclusion Criteria: Weight status: We will attempt to obtain a sample of normal weight children (age- and sex-specific BMI<85th percentile based on CDC BMI growth charts[29]) and overweight and obese children (BMI≥85th percentile). We will enroll students in 3rd-5th grades (8 to 11 y). We will invite both genders of parents to participate but anticipate the majority to be mothers. *Hispanic Origin:* This study will include all ethnicities/races; however, the selected schools are >50% Hispanic, and therefore, we expect the majority of our students and parents to be Hispanic. All school stakeholders and GLCs members will be eligible to participate.

Exclusion Criteria: Children or parents presently taking medication(s) or diagnosed with any disease that could influence dietary intake or body composition; previously diagnosed with any major illness since birth (e.g. chronic birth asphyxia, cancer, etc.); or having any physical, cognitive, or psychological disability that would prevent participation in the study will be excluded. Children testing positive for diabetes will be referred to Dr. Pont (Co-I on this study) for treatment. (*Note: these participants will still be eligible to participate in the study, and we will assess whether or not to include their data in the analyses*).

c. Benefits

There are no direct benefits to the subject, other than the ability to gain knowledge about how scientific research works. There may be no benefits to the participants. However, the TX Sprouts program will likely improve dietary intake and reduce obesity and related metabolic disorders. Participants will also benefit from the intense obesity and metabolic screening and will be notified if they have pre-diabetes or type 2 diabetes. Thus, the minimal risks that the subjects may be exposed to as a result of participating in this study appear reasonable, given the expected gains from the research.

d. Risks

Surveys and questionnaires: The survey and interview questions will collect data regarding dietary intake, dietary behaviors, and lifestyle behaviors, which are not expected to make the participant feel uneasy or embarrassed. We will have the children complete these questionnaires in a classroom. As described above, all of the procedures to be utilized involve minimal risk and discomfort to the subjects. However, each subject will be told at the onset of the task that he/she has the right to end the task whenever he/she wants to or feels distressed. All personnel will be CITI certified to work with human subjects.

Anthropometrics and body fat: To help minimize the participant from feeling self-conscious about these measures, we will perform them in either a private room or behind a privacy screen. We may also have the research staff wear white laboratory coats.

Fasting blood draw: Bilingual certified phlebotomists with at least two years of experience conducting research projects will be used for the blood collection. Symptoms are usually brief and are not serious and generally go away within a few minutes without treatment by simply having the participant lie down and rest. There is a slight, but possible risks of 1) Excessive bleeding, 2) Fainting or feeling light-headed 3) Hematoma, 4) Pain, and 5) Infection.

e. Recruitment

A flow chart depicting the study participants through the study appears in **Figure 1**. Sixteen elementary schools will be allocated to either TX Sprouts intervention (n=8 schools) or Control group (n=8 schools). The schools from the sampling frame average approximately 300 3rd-5th grade students for a total of 4,800 3rd-5th grade students. All participants and their parents will have to sign assent and consent forms, approved by UT-Austin's Institutional Review Board. Based on previous intervention work with Hispanic communities[30-32], we anticipate ~50% of parents will consent for their children to participate, and their children will complete all the baseline measures (n=2,400). We anticipate 15% attrition at post-intervention measurement due to parents/students moving away or being sick or absent from school, which would mean that 2,040 children (or 127 children per school) would complete the post-testing measures in one school year. Based on blood draw participation rates in LA Sprouts, we anticipate that 50% of participating children (n=1,200) will also participate in the blood draws at pre-intervention and 40% (n=960 or 60 per school) at post-intervention.

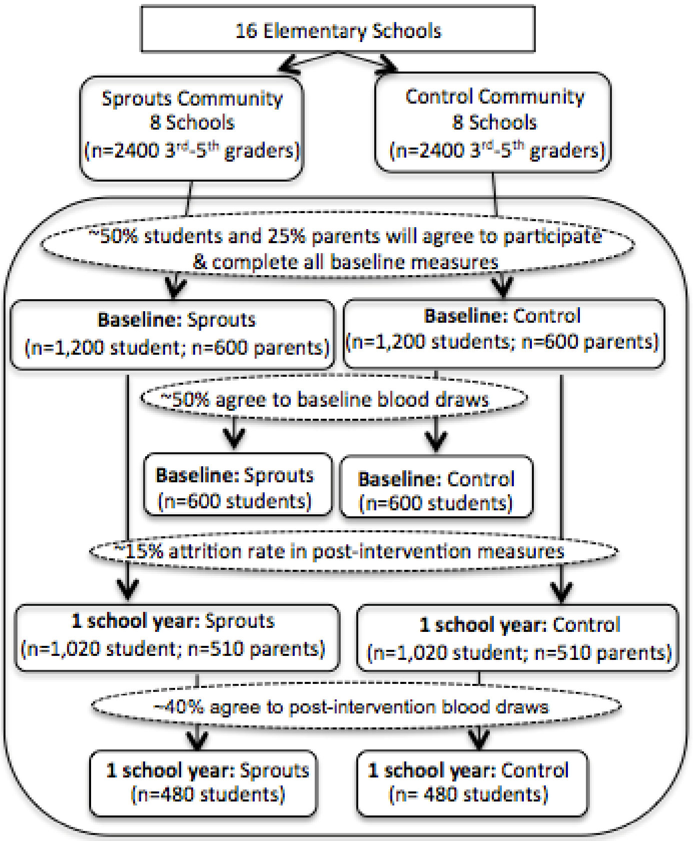

Figure 1: Flow of participants through study

Important components of our recruitment and retention strategy developed from numerous years of research with Hispanic children/families include: 1) bilingual staff, 2) Spanish and English forms/questionnaires written at a 3rd grade reading level, and 3) incentives for participation.

We will attempt to recruit approximately 10-15 school stakeholders (i.e., teachers, principals, administration staff) and 5-10 GLCs members to conduct structured interviews with over sustainability issue. The principals at each school will likely help recruit these individuals.

f. ***Obtaining Informed Consent***

The investigator will obtain signed informed consent from students and parents to collect measures before enrollment to the study. The blood draw is a voluntary measure for this study and a separate informed consent from students and parents will be collected (Appendix C). Students and parents will be recruited through a TX Sprouts Study Information Packet sent home to the parents in students' weekly homework folders. The TX Sprouts Study Information Packet will contain: an information letter, the Parental Permission, and Parental Informed Consent (Appendix D). Parents who are interested will be asked to sign the Parental Permission- Assent Form, collect the signature from their child, and complete the Parent Survey at baseline and post-intervention. They will return these documents to the child's teacher through the homework folder. Consent forms may be returned without the signature of the student. If this should occur, a research staff member will read the information to the student and collect his / her signature. Their choice not to participate in the study will be honored, when applicable. We will also set up TX Sprouts information booths at school events such as "Back to School" night, PTA meetings, and other school events where parents will be present to collect parent and student consent form signatures. We will also include information about our project in newsletters and email announcements to the parents (see recruitment flyer in Appendix E). Teachers, staff, and GLC will also be asked to sign a consent form prior to the interview or being audio recorded (see Appendix F).

7. *Privacy and Confidentiality*

Privacy will be maintained through out the experiment. The questionnaires will be filled out in the presence of only a researcher in a classroom. The Time on Task field notes will be handwritten and transferred to a computer. The contact information will be entered into a computer spreadsheet.

- Any audio and video recordings will be stored securely and only the research team will have access to the recordings. Recordings will be kept for 3 years and then erased. Only study staff will have access to this data. This original data will not be shared with other researchers.
- All blood samples will be kept in a locked freezer in Dr. Davis' laboratory. All blood samples will be associated with a code rather than their identifiable data.
- The subject data collected with the questionnaires, anthropometric measures, interview, Time on Task observation, and any contact information given on the form will be associated with a code rather than their identifiable information. The master key that can link the subject to their data will be stored in a separate, locked location and only designated research staff will have access to this file. The computer files will be password protected and only accessible by designated research staff. All identifiable data collected from this study will be stored for 3 years and then destroyed.

8. *Compensation*

A subsample of children will complete three 24-hour diet recalls and we will provide any child that completes at least two diet recalls with a $10 gift card. These gift cards will be handed out in person at their schools. If participants cannot obtain their gift cards at school, a check will be mailed home to them. Students will also receive $15 in incentives throughout the study to participate, including a study t-shirt, water bottle and cooking utensils. These incentives will be handed out during TX Sprouts lessons throughout the school year. In addition, students will receive a small toy ($1) immediately after completing the evaluation measures at baseline and post intervention. In order to get participation in the blood draws, we will offer the child a $20 cash incentive for each blood draw, which will be given immediately after the draw. We will provide the -screening results to the parents, which will be mailed to the parent, within one month of the test (See Appendix G). We have used these approaches with success in LA Sprouts. Parents will receive $15 in incentives (groceries, cooking supplies, etc.) at baseline and post-intervention for each completed questionnaire packets (pre- and post-intervention they will be compensated for completing the questionnaire). We will email, text or call parents to remind them to complete and return surveys. The incentives will be distributed to parents during the parental lessons.

References:

1. Hoelscher Dm, Springer Ae, Ranjit N, Et Al. Reductions In Child Obesity Among Disadvantaged School Children With Community Involvement: The Travis County Catch Trial. *Obesity (Silver Spring).* Feb 2010;18 Suppl 1:S36-44.
2. American Heart Association. Understanding Blood Pressure Reading. Http://Www.Heart.Org/Heartorg/Conditions/Highbloodpressure/Abouthighbloodpressure/Understanding-Blood-Pressure-Readings_Ucm_301764_Article.Jsp Last Assessed On 9/1/13.
3. Nutritionquest. Block Kids Food Frequency Questionnaire (2004). Http://Www.Nutritionquest.Com/Products. Last Accessed On May 1st, 2010.
4. Cullen Kw, Watson K, Zakeri I. Relative Reliability And Validity Of The Block Kids Questionnaire Among Youth Aged 10 To 17 Years. *J Am Diet Assoc.* May 2008;108(5):862-866.
5. Garcia-Dominic O, Trevino R, Echon R, Et Al. Improving Quality Of Food Frequency Questionnaire Response In Low-Income Mexican American Children. *Health Promotion Practice.* 2012;Doi: 10.1177/152483991140587.
6. Conway Jm, Seale Jl, Jacobes Dr, Irwin Ml, Ainsworth Be. Comparison Of Energy Expenditure Estimates From Doubly Labeled Water, A Physical Activity Questionnaire, And Physical Activity Records. *American Journal Of Clinical Nutrition.* 2002;75(3):519-525.
7. Block G, Gillespie C, Rosenbaum Eh, Jenson C. A Rapid Food Screener To Assess Fat And Fruit And Vegetable Intake. *American Journal Of Preventive Medicine.* May 2000;18(4):284-288.
8. Wardle J, Robb K, Johnson F. Assessing Socioeconomic Status In Adolescents: The Validity Of A Home Affluence Scale. *J Epidemiol Community Health.* Aug 2002;56(8):595-599.
9. Ryan Rm, Connell Jp. Perceived Locus Of Causality And Internalization: Examining Reasons For Acting In Two Domains. *J Pers Soc Psychol.* Nov 1989;57(5):749-761.
10. Williams Gc, Grow Vm, Freedman Zr, Ryan Rm, Deci El. Motivational Predictors Of Weight Loss And Weight-Loss Maintenance. *J Pers Soc Psychol.* Jan 1996;70(1):115-126.
11. Baranowski T, Davis M, Resnicow K, Et Al. Gimme 5 Fruit, Juice, And Vegetables For Fun And Health: Outcome Evaluation. *Health Educ Behav.* Feb 2000;27(1):96-111.
12. Baxter Sd, Thompson Wo. Fourth-Grade Children's Consumption Of Fruit And Vegetable Items Available As Part Of School Lunches Is Closely Related To Preferences. *J Nutr Educ Behav.* May-Jun 2002;34(3):166-171.
13. Cullen Kw, Baranowski T, Owens E, Marsh T, Rittenberry L, De Moor C. Availability, Accessibility, And Preferences For Fruit, 100% Fruit Juice, And Vegetables Influence Children's Dietary Behavior. *Health Educ Behav.* Oct 2003;30(5):615-626.
14. Domel Sb, Baranowski T, Davis H, Leonard Sb, Riley P, Baranowski J. Measuring Fruit And Vegetable Preferences Among 4th- And 5th-Grade Students. *Prev Med.* Nov 1993;22(6):866-879.
15. Reynolds Kd, Yaroch Al, Franklin Fa, Maloy J. Testing Mediating Variables In A School-Based Nutrition Intervention Program. *Health Psychol.* Jan 2002;21(1):51-60.
16. Carnell S, Wardle J. Measuring Behavioural Susceptibility To Obesity: Validation Of The Child Eating Behaviour Questionnaire. *Appetite.* Jan 2007;48(1):104-113.
17. Pate R, Ross R, Dowda M, Trost S, Sirard J. Validation Of A 3-Day Physical Activity Recall Instrument In Female Youth. *Pediatric Exercise Science.* 2003;15:257-265.
18. Mathews D, Hosker J, Rudenski A, Naylor B, Treacher D, Turner R. Homeostasis Model Assessment: Insulin Resistance And Beta-Cell Function From Fasting Plasma Glucose And Insulin Concentration In Man. *Diabetologia.* 1985;28:412-419.
19. Cruz M, Goran M. *The Metabolic Syndrome In Children And Adolescents.* Current Diabetes Reports2004.
20. Cook S, Weitzman M, Auinger P, Nguyen M, Dietz Wh. Prevalence Of A Metabolic Syndrome Phenotype In Adolescents: Findings From The Third National Health And Nutrition Examination Survey, 1988-1994. *Archives Of Pediatrics & Adolescent Medicine.* Aug 2003;157(8):821-827.
21. Donner A, Klar N. *Design And Analysis Of Cluster Randomization Trials In Health Research.* New York2010.
22. Murray D. *Design And Analysis Of Group-Randomized Trial.* New York1998.

23. Castro Dc, Samuels M, Harman Ae. Growing Healthy Kids: A Community Garden-Based Obesity Prevention Program. *American Journal Of Preventive Medicine.* Mar 2013;44(3 Suppl 3):S193-199.

24. Perez A, Dennis Rj, Gil Jf, Rondon Ma, Lopez A. Use Of The Mean, Hot Deck And Multiple Imputation Techniques To Predict Outcome In Intensive Care Unit Patients In Colombia. *Statistics In Medicine.* Dec 30 2002;21(24):3885-3896.

25. Schafer Jl. *Analysis Of Incomplete Multivariate Data.* New York1997.

26. Schafer Jl, Graham Jw. Missing Data: Our View Of The State Of The Art. *Psychological Methods.* Jun 2002;7(2):147-177.

27. Cole Da, Maxwell Se. Testing Mediational Models With Longitudinal Data: Questions And Tips In The Use Of Structural Equation Modeling. *Journal Of Abnormal Psychology.* Nov 2003;112(4):558-577.

28. Jl K, Dp M. Multilevel Modeling Of Individual And Group Level Mediated Effects. *Multivariate Behavioral Research.* 2001;36(7):249-277.

29. Centers For Disease Control And Prevention: *Cdc Growth Charts*. Atlanta, Ga, U.S. Department Of Health And Human Services, Centers For Disease Control And Prevention, National Center For Health Statistics, 2000 (U.S. Publ. No. 314).

30. Davis Jn, Tung A, Chak Ss, Et Al. Aerobic And Strength Training Reduces Adiposity In Overweight Latina Adolescents *Msse.* 2010;41(7):1494-1503.

31. Davis Jn, Kelly La, Lane Cj, Et Al. Randomized Control Trial To Reduce Obesity Related Diseases In Overweight Latino Adolescents. *Obesity.* 2009;17:1542-1548.

32. Davis Jn, Ventura Ee, Cook La, Gyllenhammer Le, Gatto Nm. La Sprouts: A Gardening, Nutrition And Cooking Intervention For Latino Youth Improves Diets And Attenuate Weight Gain. *J Am Diet Assoc.* 2011;111:1224-1230.

LIST OF APPENDICES

Appendix A: Phone Recruitment Procedure
Appendix B: Student and Parent Surveys
Appendix C: Informed Consent, Student Assent for Blood Draw
Appendix D: Student information/recruitment sheet
Appendix E: Parent Permission, Informed consent, Student Assent
Appendix F: Flyer/Announcement
Appendix G: Garden Clinical Data Sheet, Diabetes Screening Results
