# Supplementary material for: Effects of a School-Based Nutrition, Gardening, and Cooking Intervention on Metabolic Parameters in High-risk Youth: A Secondary Analysis of a Cluster Randomized Clinical Trial
Source: JAMA Netw Open. 2023 Jan 10;6(1):e2250375. doi: 10.1001/jamanetworkopen.2022.50375 (PMC9856961; doi:10.1001/jamanetworkopen.2022.50375)
Supplement: Supplement 2. — Data Sharing Statement [file jamanetwopen-e2250375-s002.pdf]

Data Sharing Statement

Davis. Effects of a School-Based Nutrition, Gardening, and Cooking Intervention on Metabolic Parameters in High-risk Youth. *JAMA Netw Open*. Published January 10, 2023. doi:10.1001/jamanetworkopen.2022.50375

Data
Data available: Yes
Data types: Deidentified participant data
How to access data: De-identified data will be available by request to Jaimie Davis (Jaimie.davis@austin.utexas.edu), and a data sharing agreement will need to be completed.
When available: With publication

Supporting Documents
Document types: Informed consent form
How to access documents: consents will be made available by request to Jaimie Davis (Jaimie.davis@austin.utexas.edu)
When available: With publication

Additional Information
Who can access the data: researchers whose proposed use of the data has been approved.
Types of analyses: For specified purposes
Mechanisms of data availability: after approval of a proposal and signed data sharing agreement
